# On Naevi and Melanomas: Two Sides of the Same Coin?

**DOI:** 10.3389/fmed.2021.635316

**Published:** 2021-02-19

**Authors:** Katie J. Lee, Monika Janda, Mitchell S. Stark, Richard A. Sturm, H. Peter Soyer

**Affiliations:** ^1^Dermatology Research Centre, The University of Queensland Diamantina Institute, The University of Queensland, Brisbane, QLD, Australia; ^2^Centre for Health Services Research, The University of Queensland, Brisbane, QLD, Australia; ^3^Department of Dermatology, Princess Alexandra Hospital, Brisbane, QLD, Australia

**Keywords:** precancer, precursor lesion, genetics and genomics, artificial intelligence, risk stratification, melanoma, naevi

## Abstract

Benign naevi are closely linked to melanoma, as risk factors, simulators, or sites of melanoma formation. There is a heavy genetic overlap between the two lesions, a shared environmental influence of ultraviolet radiation, and many similar cellular features, yet naevi remain locally situated while melanomas spread from their primary site and may progress systemically to distal organs. Untangling the overlapping contributors and predictors of naevi and melanoma is an ongoing area of research and should eventually lead to more personalized prevention and treatment strategies, through the development of melanoma risk stratification tools and early detection of evolving melanomas. This will be achieved through a range of complementary strategies: risk-adjusted primary prevention counseling; the use of lesion imaging technologies such as sequential 3D total body photography and consumer-performed lesion imaging; artificial intelligence deep phenotyping and clinical assistance; a better understanding of genetic drivers of malignancy, risk variants, clinical genetics, and polygenic effects; and the interplay between genetics, phenotype and the environment.

## Introduction

It is well-known that benign naevi and melanomas are closely linked. Many melanomas arise in or adjacent to otherwise benign naevi (1), and naevi are the most common simulators of melanoma. Number of naevi is the strongest phenotypic risk factor for melanoma, and high naevus count is associated with a younger age of melanoma onset (2, 3). A high total nevus count is also associated with a greater chance of having multiple primary melanomas, compared to single primary melanomas (4).

Not surprisingly, there is heavy genetic overlap between nevus- and melanoma-associated genes, and between the phenotypic and cellular features of naevi and melanoma, including activating mutations in oncogenes and increased proliferation rates (5). Indeed, histopathological diagnoses ranging from moderately dysplastic naevi to early stage invasive melanoma are not reproducible between different histopathologists, even though histopathological diagnosis remains the gold standard (6). Yet proliferating nevus cells migrate only locally, while melanoma cells have the capacity to spread systemically, and for any one individual nevus to transform into a melanoma is extremely rare (7).

This conundrum was the basis of our Center of Research Excellence for the Study of Naevi (CRE Naevi), a 5-year effort which aimed to advance our understanding of naevi and melanomas. The CRE teams study genetics of naevi formation, number and morphology, somatic mutations in naevi and environmental factors like UV exposure. The intention was to identify phenotypic and genetic markers that could lead to personalized genomic medicine via risk stratification, more appropriate surveillance practices, and improved health outcomes. Based in Queensland, Australia, the melanoma capital of the world (8), the CRE Naevi is in an ideal position to examine these questions, and this article will cover the state of this field at present.

Precancer (9) and preprocancer (10) concepts, which seek to determine which suspicious lesions and early cancers are likely to progress to invasive cancer, are increasing in importance. Despite the generally high sensitivity of Australian clinicians at detecting melanomas and the widespread use of dermoscopy, Australian primary care clinicians currently excise ~20 benign lesions for each melanoma, compared to 29 in other countries (11, 12). These currently-unavoidable excisions are perhaps unnecessary for cancer treatment, but provide peace of mind for clinicians and patients alike. However, being able to confidently exclude melanoma in cases where a benign nevus presents with suspicious features, and thus reduce excisions, would both benefit patients and reduce strain on the healthcare system.

On the other side of the coin, the incidence of *in situ* melanomas has also soared as detection methods become ever more refined, leading to overdiagnosis—defined as the diagnosis of lesions that are true cancers, but which would never have caused harm in the patient's lifetime (13, 14). As many as 58% of melanomas in Australia may be overdiagnosed, the majority of these being very thin *in situ* melanomas—currently indistinguishable from the very thin melanomas with invasive potential, where early excision is almost always curative (15); these figures are on top of benign naevi that are incorrectly diagnosed (or “overcalled”) by dermatopathologists as melanoma. The ability to distinguish indolent cancers from cancers with invasive potential would likewise reduce costs, adverse effects of treatments, and patient distress. Finally, studies of precursor lesions in other cancers have improved our knowledge of tumorigenesis (16), and there is every reason to suppose that nevus development can shed similar light on melanomagenesis.

In addition, technology advances in total body photography (TBP) (17) and artificial intelligence (18), and increasing access to large, high-quality, annotated image databases (19) in the last decade have opened the possibility of automated assessments of individual lesions and overall melanoma risk. Artificial intelligence-based support of clinical decision making is a particularly promising area of research (20).

## Technologies in Surveillance and Early Detection

Early diagnosis of melanoma is critical. People with a melanoma <1 mm thick at diagnosis have a 98.7% 5-year survival rate, but survival rates dive steeply with increasing thickness (21, 22). The basic detection method—clinician-led skin examinations, paying close attention to any suspicious lesion including naevi—has been greatly augmented by the widespread uptake of dermoscopy, and as reported by the Cochrane Collaboration, “visual inspection using the naked eye alone is not good enough and melanomas may be missed” (23). However, clinicians do require training in dermoscopy before they can expect to see improvements in diagnostic accuracy, and studies suggest that improvements in accuracy are more due to increases in specificity, rather than sensitivity; some featureless melanomas may still be missed (24).

However, new technologies emerging over the last decade are set to further improve both the accuracy of melanoma detection through 3D total body photography (3D TBP) and sequential dermoscopic imaging, and to extend the reach of specialist examinations to rural, remote, and underserved areas through mobile teledermatology and telehealth.

3D TBP differs from more standard 2D body photography by having the patient stand inside a matrix of cameras that all take photos at once, thus covering the body from all angles, which are then stitched together with a computer program to produce a full-body avatar on screen that can be turned around, zoomed in on, or other manipulations to allow a close look at almost any part of the skin surface. In contrast, 2D TBP requires the patient to adopt a series of anatomic poses for photography by a single camera, and these images are examined individually. While 2D TBP hardware is much cheaper than the 3D version, it is easier for individual lesions to be imaged twice or missed altogether due to the overlapping nature of the images. In both 2D and 3D imaging, there are still limitations on capturing the skin at the soles of the feet, scalp, genitals, and other folds of the body.

Sequential, 3D TBP is particularly useful for patients with many naevi or a personal or family history of melanoma, as it allows clinicians to monitor a large number of naevi for change, the number one marker of malignant transformation. Further refinement of these methods should reduce the benign to malignant excision ratio, by allowing clinicians to monitor lesions with more confidence. In addition, in our experience, study participants and patients are highly engaged by the 3D images and their use, and this may reinforce the importance of sun-protective behavior and skin self-examinations (25). TBP, including 2D photography, also seems to reduce patient anxiety about their skin in people with prior melanomas (26).

Consumer-facilitated nevus monitoring is an ongoing area of interest. More than half of all melanomas are first noticed by the patient themselves or a family member (27), and skin self-examinations are currently the recommended level of monitoring for many Australians (28). Mobile dermoscopes, partnered with apps for telehealth, may eventually be able to integrate regular skin self-examinations with teledermatology, especially for high-risk patients who need frequent monitoring; our CRE team found that such technology is feasible and acceptable to people, with further work needed to optimize lesions selection by consumers (29).

Earlier work shows that diagnostic accuracy for lesions selected by patients is similar between mobile dermoscopy and in-person examination, although further work is needed to guide people to patients reliably select concerning lesions (30, 31). Patients themselves nominate low cost, ease of access and convenience as the main factors inclining them to use mobile telehealth (32), and focus groups indicate that up to 95% of Australians would consider using an app to send images of skin lesions to a doctor, preferably for monitoring lesions between regular face-to-face appointments (33). Participant enthusiasm for mobile teledermatology decreases once they actually begin using it, but they still find it generally acceptable (34).

However, there are drawbacks to self-assessment to be addressed. For example, when asked whether they had few, some, or many naevi, 40% of participants misclassified themselves compared to a dermatologist's assessment, even with an error buffer of ±5 naevi (35). Self-imaging with a mobile device may also be limited by a participant's ability to image lesions on hard-to-reach body sites, or even to realize that those lesions might require imaging.

## Genetics and Genomics of Naevi, Melanoma and Melanoma Subtypes

Nevus count is highly heritable [heritability (h^2^) = 60–70%] (36) and most nevus-associated genes are also associated with melanoma risk, including *IRF4, MITF, MTAP*, and *PLA2G6* (37). However, not all melanoma genes affect nevus count (38). In addition, there is an overlap between somatic mutations found in naevi and melanomas, suggesting a procession of mutations necessary but not sufficient for malignant transformation that may in the future help diagnose lesions as needing monitoring but not yet excision.

### Risk Variants, Mutation Signatures, and Polygenic Effects

Genome-wide association studies (GWAS), whole genome, and whole exome sequencing have identified many melanoma risk alleles, which have a low to moderate effect individually (39). However, their effects appear to be cumulative and may add up to a significant risk (40). A nevus GWAS meta-analysis shows that variants in *MTAP, PLA2G6, IRF4, KITLG* and the 9q32 region affect nevus count (38); combining this with a meta-analysis (41) of melanoma GWAS studies showed that *GPRC5A, CYP1B1, PPARGC1B, HDAC4, FAM208B, DOCK8*, and *SYNE2* were associated with nevus count as well as melanoma risk. Recently, the number of genes identified by GWAS meta-analysis for melanoma susceptibility has expanded to 54 independent loci (42), reinforcing the importance of naevogenesis, pigmentation, telomere maintenance, and other potential pathways in the pathogenesis of melanoma (Table 1).

**Table 1:** Genes conferring susceptibility to naevi and/or melanoma.

**Gene**	**Nevus count GWAS *p*-value**	**Melanoma GWAS *p*-value**
**Low to medium penetrance** **(****38**, **43****)**		
*ASIP*	0.215	8.36 × 10^−25^
*ATM*	0.006	1.38 × 10^−12^
*CASP8*	0.134	8.88 × 10^−09^
*CDKAL1*	0.351	3.27 × 10^−08^
*CYP1B1*	5.70 × 10^−07^	2.40 × 10^−05^
*DOCK8*	1.95 × 10^−08^	5.52 × 10^−06^
*FAM208B*	1.95 × 10^−08^	5.52 × 10^−06^
*FMN1*	6.52 × 10^−06^	1.43 × 10^−06^
*FTO*	0.014	1.81 × 10^−09^
*GPRC5A*	5.72 × 10^−06^	4.08 × 10^−07^
*HDAC4*	7.59 × 10^−07^	2.16 × 10^−04^
*IRF4*	4.21 × 10^−67^	8.22 × 10^−01^
*KITLG*	8.40 × 10^−09^	6.61 × 10^−01^
*MC1R*	0.067	6.24 × 10^−92^
*MITF* ^(2)^	5 × 10^−4^	2 × 10^−18^
*MTAP* rs869329	2.12 × 10^−37^	1.14 × 10^−31^
*MTAP* rs11532907	2.30 × 10^−17^	1.42 × 10^−19^
*MX2*	0.063	3.28 × 10^−15^
*NFIC*	2.22 × 10^−04^	1.02 × 10^−05^
*OCA2*	0.935	3.11 × 10^−09^
*PARP1*	0.004	3.59 × 10^−13^
*PLA2G6* rs132985	3.06 × 10^−18^	4.76 × 10^−12^
*PLA2G6* rs2005974	3.31 × 10^−14^	7.83 × 10^−11^
*PPARGC1B*	4.71 × 10^−07^	4.58 × 10^−04^
*SETDB1*	0.007	3.88 × 10^−12^
*SLC45A2*	0.755	2.30 × 10^−12^
*SYNE2*	1.95 × 10^−04^	1.74 × 10^−05^
*TCONS_12_00025686*	0.002	1.84 × 10^−09^
*TERC*	5.73 × 10^−06^	1.64 × 10^−05^
*TERT*	0.003	1.66 × 10–^17^
*TPCN2/CCND1*	0.209	1.01 × 10^−10^
*TTC7B*	0.415	4.63 × 10^−14^
*TYR*	0.605	1.01 × 10^−26^
*9q31* region	1.35 × 10^−04^	2.30 × 10^−08^
**High-penetrance familial melanoma** **(****44****)**		
*ACD*		+
*ACH*		+
*BAP1*		+
*CDK4*		+
*CDKN2A*	+	+
EBF3^*^		+
GOLM1^*^		+
NEK11^*^		+
POLE^*^		+
*POT1*		+
*TERF2IP*		+
*TERT*	+	+

* *Proposed high-penetrance melanoma genes; further research to confirm these is ongoing*.

+ *Indicates association*.

The effect of nevus-related genes on melanoma risk can be multiplied by polygenic effects for genes controlling other phenotypic risk factors. For example, *MC1R* “R” variants are best known for causing the red hair phenotype and have only a small effect on nevus count (43). However, R alleles and high nevus counts synergistically increase melanoma risk (45–47). People with *MC1R* wildtype (WT) genotype and 20+ naevi have a similar melanoma odds ratio (OR) to people homozygous for the *MC1R* R allele (R/R) with 0–4 naevi (OR 4.82 vs. 4.42, respectively.) In contrast, people with both risk factors—an R/R genotype and 20+ naevi—have a melanoma OR of 25.09 (47).

Genetic variations also influence the risk of having more than one primary melanoma. For example, the E318K variant in *MITF*, combined with *MC1R* R alleles and other haplotypes in e.g., *ASIP*, were all at elevated frequencies in people with multiple primary melanomas compared to people with a single primary melanoma (4). Polygenic risk scores, accounting for the cumulative risk of many low- to moderate-effect melanoma-associated alleles (40), are higher in multiple primary melanoma than single primary melanoma or no melanoma history participants (4, 48).

Various melanoma subtypes are also associated with particular genetic variants. For example, amelanotic and hypomelanotic melanomas are associated with the *MTAP* and *PLA2G6* alleles associated with overall melanoma risk, nevus morphology and high nevus count; however, amelanotic/hypomelanotic melanoma patients do not have a significantly different total nevus count from those with the more common pigmented melanomas (49). This observation may be due to the older age at diagnosis of amelanotic/hypomelanotic melanoma compared to pigmented melanoma (50–52), as nevus count declines from the fourth decade onwards (53). Albinism-related genes such as *TYR* and *OCA2* have deleterious variants that are also more common in patients with amelanotic/hypomelanotic melanoma (54).

Several melanoma/nevus genes have multiple associations to phenotypes. An example is the *IRF4* intronic variant rs12203592^*^T, which is associated with increased melanoma risk, increased risk of nodular melanoma (55), increased Breslow thickness at diagnosis (56), high nevus count in childhood and low nevus count in adulthood (57), and globular dermoscopic subtype of naevi (43, 58). This SNP is associated with pigmentary phenotype of darker hair color, lighter eyes, and skin with a lower tanning response to sunlight (37). The different alleles exhibit differential expression levels that also influence a range of key immunomodulatory molecules and cytokines, and melanocyte growth and survival after UV exposure (59). Research continues on untangling which of these many effects contribute to nevus and/or melanoma formation and behavior (43, 60).

### Clinical Genetics and Familial Melanoma

Approximately 5–10% of melanomas arise in a familial melanoma context. While there are a handful of well-established, rare, high penetrance mutations, such as deleterious variants of *CDKN2A, CDK4, BAP1, TERT, POT1, TERF2IP, ACD, POLE, EBF3, GOLM1*, and *NEK11* associated with familial melanoma, these mutations account for only 30% of familial melanoma cases (44). Research is increasingly focusing on the 60% of familial melanoma that is not explained by these high penetrance mutations, and recent work shows that familial cluster without a high penetrance mutation are enriched for polygenic risk (48).

In the absence of an identifiable mendelian mutation, assessment of candidate risk genes and improvements in genotype-phenotype correlations, together with polygenic risk scores (4) will help identify people who are nevertheless at high risk. By assessing the cumulative risk of 21 gene regions associated with a low or moderate melanoma risk, Cust et al. (40) showed that, in a group of melanoma patients and control participants who all had a low phenotypic risk profile, 9–21% had a high polygenic risk.

### Mechanisms/Drivers of Benign Neoplasms, Malignancy, and Metastasis

The activation of the MAPK pathway is the first, essential step for melanocytic proliferation, but it is not sufficient for full malignant transformation (61). Either *BRAF* or *NRAS*, but rarely both, are mutated in almost all acquired melanocytic naevi (62, 63). In contrast, blue naevi and Spitz naevi often have *GNAQ* and *HRAS* mutations. *GNAQ* is also associated with uveal melanomas (64). This may indicate that *GNAQ/HRAS* are not key players in melanomagenesis, unlike *BRAF* and *NRAS* (64). *BRAF* mutations are also associated more strongly with the globular dermoscopic subtype than reticular naevi (92 vs. 67%) (63). Dermoscopic subtypes refer to the distribution of pigment visible in a dermoscopic image of a lesion, and correlated closely to histopathological structures, allowing a visual assessment of potentially pathological processes (64).

Most cutaneous melanomas fall into one of four categories based on their initial driver mutation: *BRAF, NRAS, NF1* and triple wild-type, which lacks any of the three other drivers (65, 66). *BRAF* V600 mutations are found in 35–50% of melanomas, and V600 variants are also found in 67–100% of benign naevi (63).

*TERT* promotor mutations, found in 86% of cutaneous melanomas and also detected in intermediate melanocytic lesions, are an early secondary alteration necessary for malignant transformation leading to melanoma development and proliferation (64, 67). The aberrant *TERT* gene leads to lengthening of the telomeres, which is not recognized as abnormal by the cell and as such apoptosis or senescence does not occur. Now that the melanocyte has lost its ability to undergo apoptosis, the series of events that occur next are likely to be related to chromosomal instability and DNA copy number aberrations. The precise order of these events has not been determined for each aberrant melanocyte, but in a study by Shain et al. (68), it was found that UV-induced point mutations continued to increase, and invasive melanoma was associated with increased copy-number aberrations, particularly loss of *CDKN2A*. Further mutations and/or genomic aberrations (e.g., copy number gain/loss and promotor methylation) are necessary for the transition of locally invasive melanoma to metastatic disease and have been described in *PTEN, AKT3, CDKN2A, CCND1, CDK4, RB1, TP53, MDM2*, and *ARID2* (Table 2) (65, 66, 70).

**Table 2:** Driver mutations and the transition to invasive and metastatic disease (65, 66, 69).

**Genes**	**Gene type**	**Mutation type**
*BRAF* (2)	Oncogene	Point mutation, translocations, copy number alteration
*NRAS* (2)	Oncogene	Point mutation, copy number alteration
*NF1* (2)	Tumor suppressor	Point mutation
*TERT*	Telomere maintenance	Point mutation, copy number alteration
*GNAQ*	Oncogene	Point mutation
*HRAS*	Oncogene	Point mutation, indel
*PTEN*	Tumor suppressor	Point mutation, epigenetic silencing, copy number alteration
*AKT3*	Oncogene	Structural rearrangement
*CDKN2A*	Tumor suppressor	Point mutation, methylation silencing, copy number alteration
*CCND1*	Oncogene	Copy number alteration
*CDK4*	Oncogene	Copy number alteration
*RB1*	Tumor suppressor	Point mutation
*TP53*	Tumor suppressor	Point mutations, copy number alteration
*MDM2*	Oncogene	Copy number alteration
*ARID2*	Tumor suppressor	Point mutations, copy number alteration

### Interplay Between Genetics, Phenotype and Environment

While excessive UV exposure is well-known to promote melanomagenesis, a significant proportion of melanomas do form on sun-protected body sites. In a recent study, 75% of those diagnosed at ≤40 years old had one or more melanomas in a UV-protected site; in participants aged >40 years, only 18% of their melanomas were in UV-protected sites (4).

To explain this, the divergent pathway model of melanoma development posits that melanomas are caused either by a UV damage-related pathway or are secondary to inherent traits such as high nevus count (71). However, it appears that both pathways are at work in many melanoma cases, with genetic and phenotypic risk factors enhanced by a high-UV environment (72). For example, while nevus count is largely genetically controlled, increased UV exposure is associated with increased nevus count in those who are genetically predisposed (71). Usually only minimal or intermittent sun-exposure is required to activate the growth of aberrant melanocytes. It can be theorized that the *BRAF* mutation is already present in skin melanocytes scattered across the body surface (73) and that UV exposure contributes to the activation and increased proliferation of melanocytes harboring the *BRAF* mutation, leading to a nevus.

A study of genetic variations in melanoma patients who had all their melanomas in visibly sun-damaged sites found that these people were more likely to have variants at *MC1R* rs75570604 (OR 2·5), *9q31.2* rs10816595 (OR 1·4), and *MTAP* rs869329 (OR 1·4), compared to people with melanomas on both sun-exposed and sun-protected sites. Interestingly, these same variants were more common in people first diagnosed ≤40 years old than those diagnosed later in life (4).

Melanomas on sun-protected body sites are more likely to arise in an existing nevus (1), suggesting a link between defects in DNA repair and both nevus and melanoma formation. However, defects in DNA repair also appear to predispose to higher rates of UV damage on the sun-exposed sites of the body. A study of naevi on participants living in the high-UV environment of Queensland, Australia, found that sun-exposed lesions contained UV-related (signature 7) somatic mutations, while naevi on sun-protected sites had a higher proportion of mutation signatures associated with defective DNA repair, particularly indels associated with defective mismatch repair (74). This pattern was also observed in a lower-UV environment Spanish cohort (75). This suggests that defective DNA repair in melanocytes provides the necessary environment for both nevus formation and the accumulation of UV damage mutations.

Whole exome sequencing shows that benign naevi have a lower mutational burden than melanomas, particularly a lower level of UV-associated mutation signatures and melanoma driver mutations (76), although C>T transitions are still the most common SNV class (74). A WES study of 30 naevi and adjacent non-lesional skin identified UV-associated signatures in 97% of nevus samples, age-related signatures in 93%, and defective DNA repair signatures in 30%. This was reversed in the adjacent non-lesional skin, despite being exposed to the same level of UV as the naevi, with UV-related signatures in 10%, defective DNA repair signatures in 83% (signature 3) and 50% (signature 26), and age-related signatures in 100% of samples. In addition, two lesions in the study had low UV-associated and no age-related signatures, highlighting the importance of defective DNA mismatch repair in the development of at least some naevi (74).

Different dermoscopic patterns of naevi are also associated with different genomic signature; globular naevi have a higher proportion of C>T transitions, while reticular naevi have a higher proportion of copy number aberrations and indels. However, without longitudinal monitoring, it is difficult to know whether these differences reflect the patient's age at nevus onset, or reflect specific mutation types driving the development of specific dermoscopic patterns (74).

Even melanocytes in seemingly normal skin have numerous pathogenic somatic mutations, although the mutation load is even higher in melanocytes from skin adjacent to a skin cancer, where it is comparable to the mutational burden in melanoma cells. As might be expected, melanocytes from sun-protected sites have fewer somatic mutations than those from chronically sun-exposed sites, but melanocytes from intermittently sun-exposed sites, such as the thigh, have even more somatic mutations than either. Recent work has found that the majority of mutations are predicted to affect the MAPK pathway, including *BRAF, NRAS, MAP2K1, NF1, CBL*, and *RASA2* (77). Other mutated genes included *CDKN2A, ARID2, PTEN*, and *DDX3X*.

## Conclusions and Perspectives

Although recent advances have shed much light on the mechanisms linking naevi and melanoma, many questions remain. In particular, imaging, genetics and genomics, and cognitive computing have enormous potential to stratify risk categories and personalize skin cancer prevention and early detection, but protocols for integrating them into regular clinical care are still in the developmental stage.

TBP with sequential digital dermoscopy or pseudo-dermoscopy, supported by self-imaging with mobile devices during skin self-examinations, will likely become the new standard to facilitate early detection and prevent advanced and metastatic disease. Research will focus on designing and validating appropriate protocols and monitoring intervals, including tailored protocols for patients at different risk levels. This work will continue to investigate the natural history of naevi across the lifetime of individuals, an area still poorly understood, since most studies of naevi are cross-sectional rather than longitudinal (78).

TBP will be further augmented with cognitive computing (20), as artificial intelligence algorithms are developed to provide triaging, clinical decision support and risk assessment based not only on nevus imaging, but also deep phenotyping. Deep phenotyping is the process of assessing the whole skin surface for phenotypic signs of genetic susceptibility, namely freckling and nevus number, type and distribution, and signs of environmental risk, by assessing the amount of UV damage over large areas of skin. These should improve our overall assessment of melanoma risk by improving the objective assessment of accumulated sun damage and the visible effects of genetic risk factors.

Genomics remains a fruitful area of research, with polygenic risk scores (40) and the identification and characterization of rare candidate risk genes (38) a particularly promising avenue of research. A combination of further discoveries of nevus- and melanoma-associated genes and regulatory regions with polygenic scores incorporating many small-to-moderate effect risk variants, as well as the well-known larger-effect variants, will eventually allow for risk stratification that directs more appropriate surveillance protocols for people with different levels of melanoma risk.

Finally, it will be critical to develop algorithms able to capture all the relevant phenotypic and genotypic factors and synthesize them into a complete risk-stratification tool to allow risk-adjusted surveillance that detects melanoma early while minimizing unintended harms of surveillance.

## Data Availability Statement

The original contributions presented in the study are included in the article/supplementary material, further inquiries can be directed to the corresponding author.

## Author Contributions

KL wrote the first draft of the manuscript. MJ, MS, RS, and HS wrote sections of the manuscript. All authors contributed to manuscript revision and read and approved the submitted version.

## Conflict of Interest

HS is a shareholder of MoleMap NZ Limited and e-derm consult GmbH, and undertakes regular teledermatological reporting for both companies. HS has NHMRC partnership grants with Canfield Scientific Inc. (APP1153046) and Fotofinder Systems Inc. (APP1113962). HS provides medical consultant services for Canfield Scientific Inc., First Derm by iDoc24 Inc, and Revenio Research Oy. HS is a board member of Melanoma and Skin Cancer Trials Limited and the QLD Skin & Cancer Foundation. HS is a member of the Australian Academy of Health and Medical Sciences Reports committee. The remaining authors declare that the research was conducted in the absence of any commercial or financial relationships that could be construed as a potential conflict of interest.
